# The Attenuation Value Within the Non-hypodense Region on Non-contrast Computed Tomography of Spontaneous Cerebral Hemorrhage: A Long-Neglected Predictor of Hematoma Expansion

**DOI:** 10.3389/fneur.2022.785670

**Published:** 2022-04-08

**Authors:** Yong Chen, Dan Cao, Zheng-Qian Guo, Xiao-Ling Ma, Yi-Bo Ou, Yue He, Xu Chen, Jian Chen

**Affiliations:** ^1^Department of Neurosurgery, Tongji Hospital, Tongji Medical College, Huazhong University of Science and Technology, Wuhan, China; ^2^Department of Radiology, Tongji Hospital, Tongji Medical College, Huazhong University of Science and Technology, Wuhan, China

**Keywords:** attenuation value, non-hypodense region, hematoma expansion, Hounsfield units, non-contrast computed tomography, spontaneous intracerebral hemorrhage

## Abstract

**Background and Purpose:**

The ability of attenuation value of the non-hypodense region of hematoma in non-contrast computed tomography (NCCT) for predicting hematoma expansion (HE) remains unclear. Our purpose is to explore this relationship.

**Methods:**

Two cohorts of patients were collected for analysis. The region where we measured hematoma attenuation values was limited to the non-hypodense region that was not adjacent to the normal brain tissue on NCCT. The critical attenuation value was derived *via* receiver operating characteristic (ROC) curve analysis in the derivation cohort and its predictive ability was validated in the validation cohort. Independent relationships between predictors, such as critical attenuation value of the non-hypodense region and HE were analyzed using the least absolute shrinkage and selection operator (LASSO) regression and multivariate logistic analysis.

**Results:**

The results showed that the attenuation value <64 Hounsfield units (HU) was independently associated with HE [odds ratio (*OR*), 4.118; 95% confidential interval (*CI*), 1.897–9.129, *p* < 0.001] and the sensitivity, specificity, positive predictive value (PPV), negative predictive value (NPV), positive likelihood ratio (PLR), negative likelihood ratio (NLR), and area under the curve (AUC) for predicting HE were 36.11%, 81.71%, 1.97, 0.78, 44.8%, 75.7%, and 0.589, respectively.

**Conclusions:**

Our research explored and validated the relationship between the attenuation value of the non-hypodense region of hematoma and HE. The attenuation value < 64 HU was an appropriate indicator of early HE.

## Introduction

**Figure 1:**
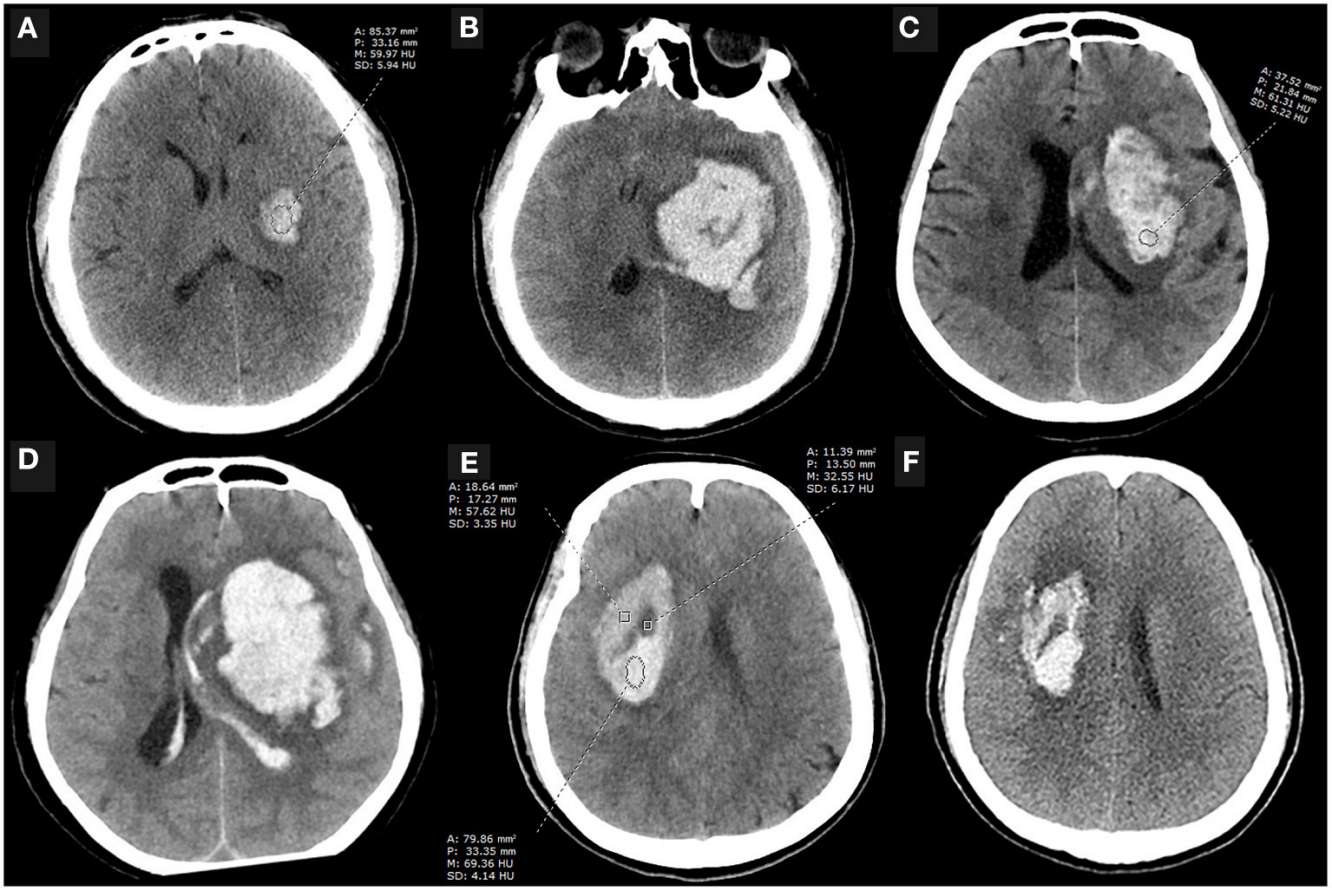
Case examples. **(A,B)** An initial NCCT scan performed 3.5 h after the onset of symptoms showed a small, regular shape hematoma with an attenuation value of 61.72 HU at the internal capsule, which enlarged 10 h later. The patient eventually died. **(C,D)** An NCCT scan performed 0.5 h after symptom onset showed a basal ganglia hematoma that was heterogeneous with an attenuation value of 63.86 HU within the non-hypodense region and appeared significantly expansion 12 h later. **(E,F)** NCCT scan performed 1 h after onset showed a heterogeneous basal ganglia hematoma with an attenuation value of more than 64 HU in the non-hypodense region, and the patient did not present with HE despite the presence of the black hole sign on the initial NCCT examination. A, area; P, perimeter; M, mean; SD, standard deviance; HU, Hounsfield units; NCCT, non-contrast computed tomography.

Hematoma expansion (HE) occurs in approximately one-third of patients with spontaneous intracerebral hemorrhage, and it is an independent risk factor for the worsening prognosis and early death (1). Early recognition of HE is considered as one of the potential therapeutic goals to improve prognosis (2, 3). In addition to the spot sign and its density on contrast CT (4), the presence of hypodense areas within the hematoma on non-contrast computed tomography (NCCT) is an important factor in assessing the inefficient clot contraction and the instability of intracerebral hematomas with a high discriminating ability (5). However, the hypodense foci on NCCT do not always match the location of the spot sign suggestive of contrast leakage on contrast CT (6), which may be implicated in the relatively low sensitivity of the NCCT signs in predicting HE (7–9). Minimally invasive surgery appeared to reduce the poor prognosis of patients at high risk of hematoma expansion, yet this difference was not significant for reasons that cannot be ruled out due to the inability to accurately identify the actual hematoma that will undergo expansion (10). In our clinical work, we have noticed that some homogeneous hematomas dilated (>6 ml or 33% increase compared with baseline volume) without any hypodensity foci but with overall low attenuation value, some dilated heterogeneous hematomas (those with a swirl sign, black hole sign, or blend sign) with relatively low attenuation value within the non-hypodense region, while some non-dilated heterogeneous hematomas have relatively high attenuation values within the non-hypodense region (Figure 1), prompting us to wonder that whether low attenuation value of the non-hypodense region is a risk factor for HE. Therefore, this study aims to investigate the clinical significance of the attenuation value within the non-hypodense region for predicting HE.

## Materials and Methods

### Patients

Patients with spontaneous intracerebral hemorrhage admitted to our three hospital branches from January 2013 to June 2021 were selected for this retrospective study. Inclusion criteria for eligible patients were (1) the first NCCT examination was performed within 6 h after onset and (2) one or more NCCT re-examinations were performed within 72 h after onset. Exclusion criteria were (1) patient age <18 years old; (2) intracerebral hemorrhage secondary to arteriovenous malformation, aneurysm, trauma, tumor, Moya-Moya disease, or other diseases; (3) multiple cerebral hemorrhages or primary ventricular hemorrhage; (4) any form of neurosurgery performed before the first NCCT re-examination; (5) patients with axial layers <3 on the first NCCT scan (aiming to eliminate partial volume effects when extracting hematoma attenuation values); and (6) baseline hematoma volume <1 ml.

### Derivation Cohort

Eligible patients admitted in the main hospital site from January 2013 to August 2015 were included as a derivation cohort according to the inclusion and exclusion criteria described above. Attenuation value within the non-hypodense region of the hematoma was examined retrospectively for suitability to predict HE, and a cutoff value was determined by an ROC analysis based on the maximum Youden index.

### Validation Cohort

Patients admitted in the main hospital site between September 2015 and June 2021 (*n* = 119) and two other branch sites (October 2015 to June 2021, *n* = 70; November 2017 to June 2021, *n* = 58) who met the above criteria were included as a validation cohort. A validating analysis was performed to investigate whether a critical attenuation value of the non-hypodense region could predict HE in this cohort. The studies involving human participants were reviewed and approved by the local ethics committee. Written informed consent was not required for this study due to the de-identified retrospective data.

### Clinical Information

Clinical information for each patient is collected from the electronic medical records, such as demographic characteristics, medical history, physical examination items, and potentially relevant laboratory tests at the time of admission (Table 1).

**Table 1:** Comparison of clinical and radiological characteristics and the outcome of derivation and validation cohort patients.

**Variables**	**Derivation cohort (*n* = 132)**	**Validation cohort (*n* = 247)**	***P***
**Clinical characteristics**			
Age, years, mean (SD)	55.0 (11.1)	57.6 (12.4)	0.039
Sex, male (%)	88 (66.7)	162 (65.6)	0.922
Hypertension (%)	81 (61.4)	171 (69.2)	0.152
Diabetes (%)	13 (9.85)	21 (8.50)	0.804
Current smoker (%)	50 (37.9)	46 (18.6)	<0.001
Current alcohol drinker (%)	36 (27.3)	42 (17.0)	0.026
Stroke history (%):			0.812
No	117 (88.6)	210 (85.0)	
Hemorrhage	6 (4.55)	17 (6.88)	
Infarction	8 (6.06)	17 (6.88)	
Hemorrhage and infarction	1 (0.76)	3 (1.21)	
Antiplatelets or anticoagulants therapy (%)	5 (3.79)	11 (4.45)	0.969
Systolic pressure on admission (mmHg), median [IQR]	162 [146; 178]	160 [144; 180]	0.807
Diastolic pressure on admission (mmHg), mean (SD)	92.4 (16.9)	92.9 (15.5)	0.795
Baseline GCS score (%):			0.519
12–15	51 (38.6)	109 (44.1)	
9–11	52 (39.4)	84 (34.0)	
3–8	29 (22.0)	54 (21.9)	
RBC count (*10^12^/L), median [IQR]	4.64 [4.37; 4.95]	4.62 [4.28; 5.00]	0.447
Hemoglobin (g/L), median [IQR]	140 [129; 150]	139 [129; 151]	0.475
Hematocrit (%), median [IQR]	41.6 [38.7; 43.7]	41.2 [38.7; 44.3]	0.700
MCV (fl), median [IQR]	89.1 [86.7; 91.4]	89.9 [87.0; 92.5]	0.237
MCH (pg), median [IQR]	30.3 [29.5; 31.2]	30.5 [29.2; 31.4]	0.917
MCHC (g/L), median [IQR]	338 [330; 348]	338 [328; 345]	0.311
RDW (%), median [IQR]	13.0 [12.5; 13.7]	12.9 [12.2; 13.6]	0.186
Platelet count (*10^9^/L), median [IQR]	196 [158; 227]	206 [171; 249]	0.082
Platelet distribution width (%), median [IQR]	13.9 [12.2; 15.8]	12.7 [11.1; 14.3]	<0.001
Prothrombin time (seconds), median [IQR]	13.4 [13.0; 13.9]	13.3 [12.9; 13.8]	0.060
Activated partial thromboplastin time (seconds), median [IQR]	34.2 [32.0; 36.8]	34.5 [32.2; 37.2]	0.434
International normalized ratio, median [IQR]	1.03 [1.00; 1.08]	1.02 [0.97; 1.06]	0.020
Total cholesterol (seconds), median [IQR]	4.27 [3.65; 5.04]	4.33 [3.70; 4.94]	0.806
Serum glucose (mmol/L), median [IQR]	6.72 [5.56; 8.43]	6.84 [5.80; 8.33]	0.640
Time from first NCCT scan to onset (hours), median [IQR]	3.00 [1.88; 4.12]	3.00 [1.50; 4.00]	0.472
**Radiological characteristics**			
Hematoma location (%):			<0.001
Thalamus	28 (21.2)	43 (17.4)	
Basal ganglia	101 (76.5)	157 (63.6)	
Brain stem or cerebella	0 (0.0)	11 (4.5)	
Cerebral lobe	3 (2.3)	36 (14.6)	
Baseline hematoma volume (ml), median [IQR]	19.6 [9.25; 34.7]	16.8 [8.69; 30.6]	0.187
Largest hematoma width/length ratio on axial section (>0.6), (%)	65 (49.2)	119 (48.2)	0.929
Midline shift distance (>0.5 cm), (%)	41 (31.1)	51 (20.6)	0.033
Subarachnoid hemorrhage (%)	7 (5.30)	15 (6.07)	0.940
Intraventricular hemorrhage (%)	48 (36.4)	62 (25.1)	0.029
Swirl sign (%)	19 (14.4)	44 (17.8)	0.479
Black hole sign (%)	10 (7.58)	26 (10.5)	0.454
Blend sign (%)	12 (9.1)	49 (19.8)	0.011
Irregular sign (%)	51 (38.6)	95 (38.5)	1.000
Satellite sign (%)	39 (29.5)	68 (27.5)	0.768
Island sign (%)	13 (9.85)	23 (9.31)	1.000
Attenuation value of non-hypodense region (HU), median [IQR]	66.2 [63.1; 69.3]	67.6 [64.4; 70.8]	0.011
**Outcome**			
HE (%)	31 (23.5%)	72 (29.1%)	0.289
3 months mRS score (4~6), (%)	60 (45.5%)	128 (51.8%)	0.283

*SD, standard deviation; IQR, interquartile range; GCS, Glasgow coma scale; RBC, red blood cell; MCV, mean corpuscular volume; MCH, mean corpuscular hemoglobin; MCHC, mean corpuscular hemoglobin concentration; RDW, red blood cell distribution width; NCCT, non-contrast CT; HU, Hounsfield units; HE, hematoma expansion; mRS, modified Rankin scale*.

### Imaging Characteristics

**Figure 2:**
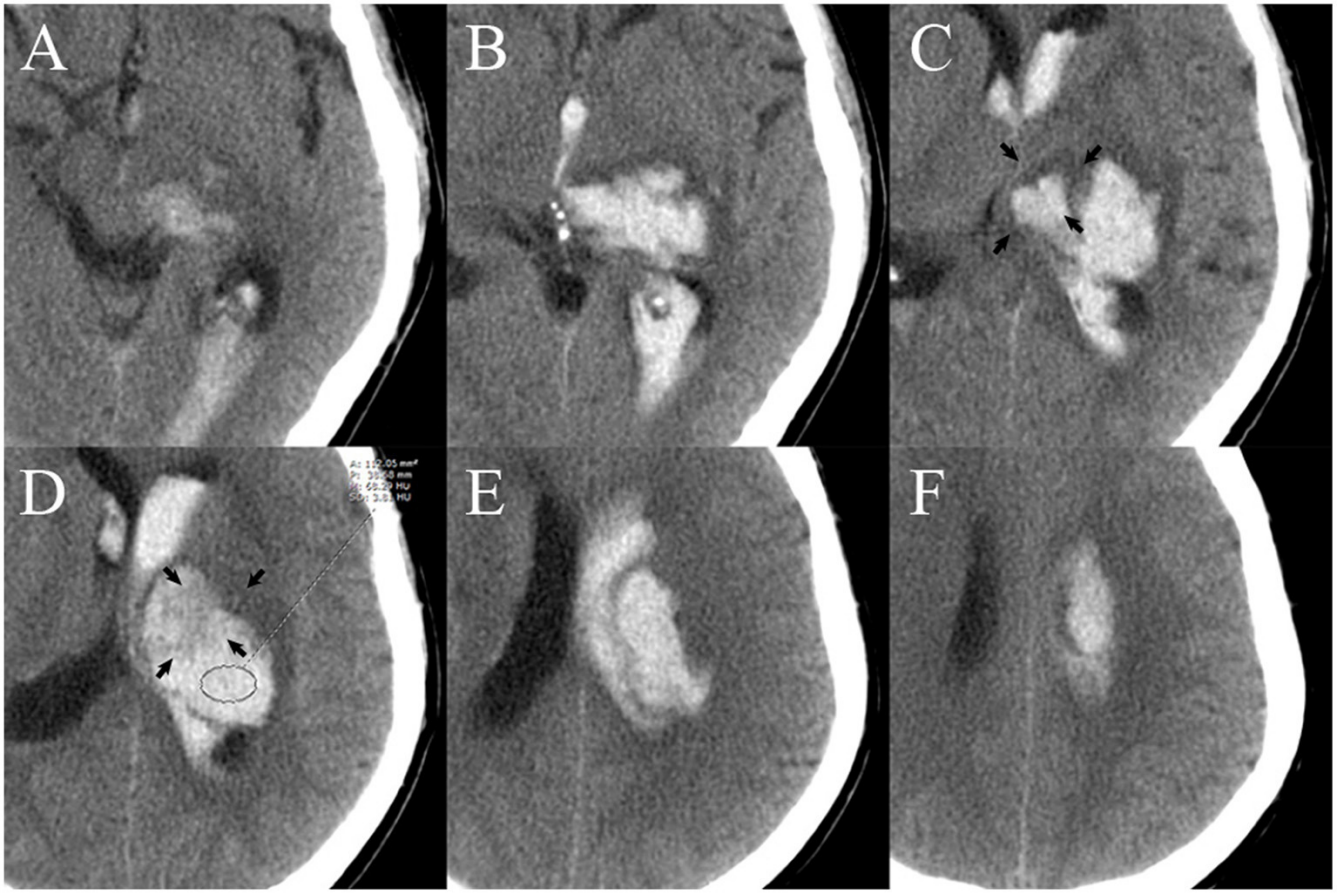
Measurement of the attenuation value of the region of interest (ROI) of the non-hyperdense region of the hematoma. There are six layers of hematoma on axial NCCT images. The upper **(A,B)** and lower layers **(E,F)** are not suitable for measurement because they are adjacent to normal brain tissue. The core two layers **(C,D)** are selected as the best layer for measurement. The area of layer **(D)** (black arrow) that is adjacent to normal tissue [layer **(C)**, black arrow] may cause partial volume effects that affect the accuracy of the measurement and is therefore excluded from the ROI. A, area; P, perimeter; M, mean; SD, standard deviance; HU, Hounsfield units; NCCT, non-contrast computed tomography.

**Figure 3:**
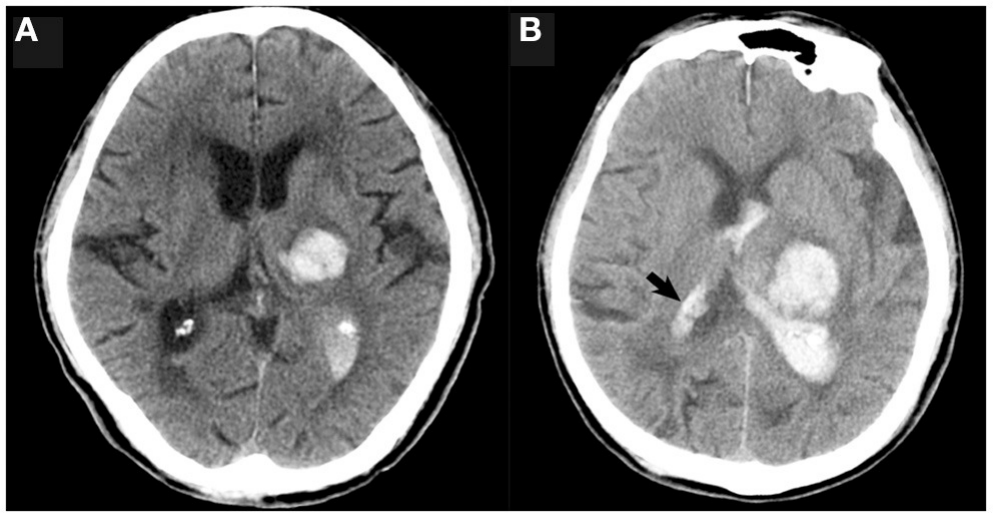
The initial NCCT showed that the thalamic hematoma broke into the ipsilateral ventricle only **(A)**; a follow-up NCCT **(B)** showed new hematoma formation in both the ipsilateral and contralateral ventricles (black arrow).

Non-contrast computed tomography images of the patients were obtained by standard clinical protocols (120 kV, axial section 5–7.5 mm thick). Baseline hematoma volume was calculated *via* the Tada formula ABC/2. The NCCT image data with DICOM format of each patient were used to measure the attenuation value of the non-hypodense region of the hematoma in the Picture Archiving and Communication System (PACS). The non-hypodense region was restricted to any layer within the highest density region of the heterogeneous hematoma as well as the core region of the homogeneous hematomas. When the standard deviance of the mean HU value in the region of interest of the non-hyperdense region is ≤ 6, this part of the hematoma is considered to be homogeneous. The layer used to measure the attenuation value was limited to the core axial section of the hematoma, and the upper and lower layers adjacent to the normal brain tissue were not used for measurement (Figure 2). The attenuation value of the hematoma was assessed independently by two experienced raters (YC and DC) who were unaware of the outcome of patient. The midline shift distance was defined as the maximum lateral vertical displacement distance of brain tissue structures in the horizontal plane from the mid-axis sagittal plane of the NCCT scan. The definitions of irregular sign, satellite sign, island sign, swirl sign, black hole sign, and blend sign were conformed to the standards that were proposed by Andrea Morotti et al. (11). HE or dilated hematoma was defined as a >33% or >6 ml increase of hematoma volume or new intraventricular hematoma development on the NCCT re-examination (Figure 3) (12).

### Statistical Analysis

Statistical analyses were performed with R software (version 4.0.5, http://www.Rproject.org) and the SPSS package (version 24.0, IBM Corporation, Armonk, NY). Categorical variables were expressed as percentages (%) and continuous variables were expressed as means [±standard deviation (SD)] or medians (interquartile range, IQR). Cohen's κ-test was used to determine inter- and intra-rater agreement referring to the NCCT signs and attenuation value level. A univariate analysis was performed using chi-square test, Fisher's exact test, two-tailed Student's *t*-test, or univariate logistic analysis, as appropriate. An ROC curve analysis with Delong's test was used to obtain cutoff for attenuation value within the non-hypodense region of the hematoma and to obtain the sensitivity, specificity, PPV, NPV, PLR, NLR, and AUC values. Considering the possible collinearity between variables, the least absolute shrinkage and selection operator (LASSO) regression was first used to screen the potential predictors of HE (13), and then variables with non-zero coefficients were further included in the multivariate model. The model was visualized using the nomogram and the discrimination and calibration of the prediction were observed by the use of Harrell's concordance index (C-index)/AUC of ROC analysis and calibration plot. The decision curve analysis and clinical impact curve analysis were used to observe the performance of the attenuation value within the non-hypodense region in the terms of improving the predictive power of the model. A two-tailed test of *p* < 0.05 was considered statistically significant.

## Results

### Clinical and Radiological Characteristics and Outcomes of Two Cohorts

In the primary and validation cohorts, 31 of 132 patients (23.5%) and 72 of 247 patients (29.1%) experienced HE. The median attenuation value of the non-hypodense region of hematoma was 66.2 HU [IQR, 63.1–69.3 HU] in the derivation cohort and 67.6 HU [IQR, 64.4–70.8 HU] in the validation cohort. Supplementary Figure 1 shows the patient selection process for both cohorts. The clinical and radiological characteristics and the outcomes of two cohort patients are shown in Table 1.

### Factors Associated With the Attenuation Value of the Non-hypodense Region of the Hematoma in the Validation Cohort

**Figure 4:**
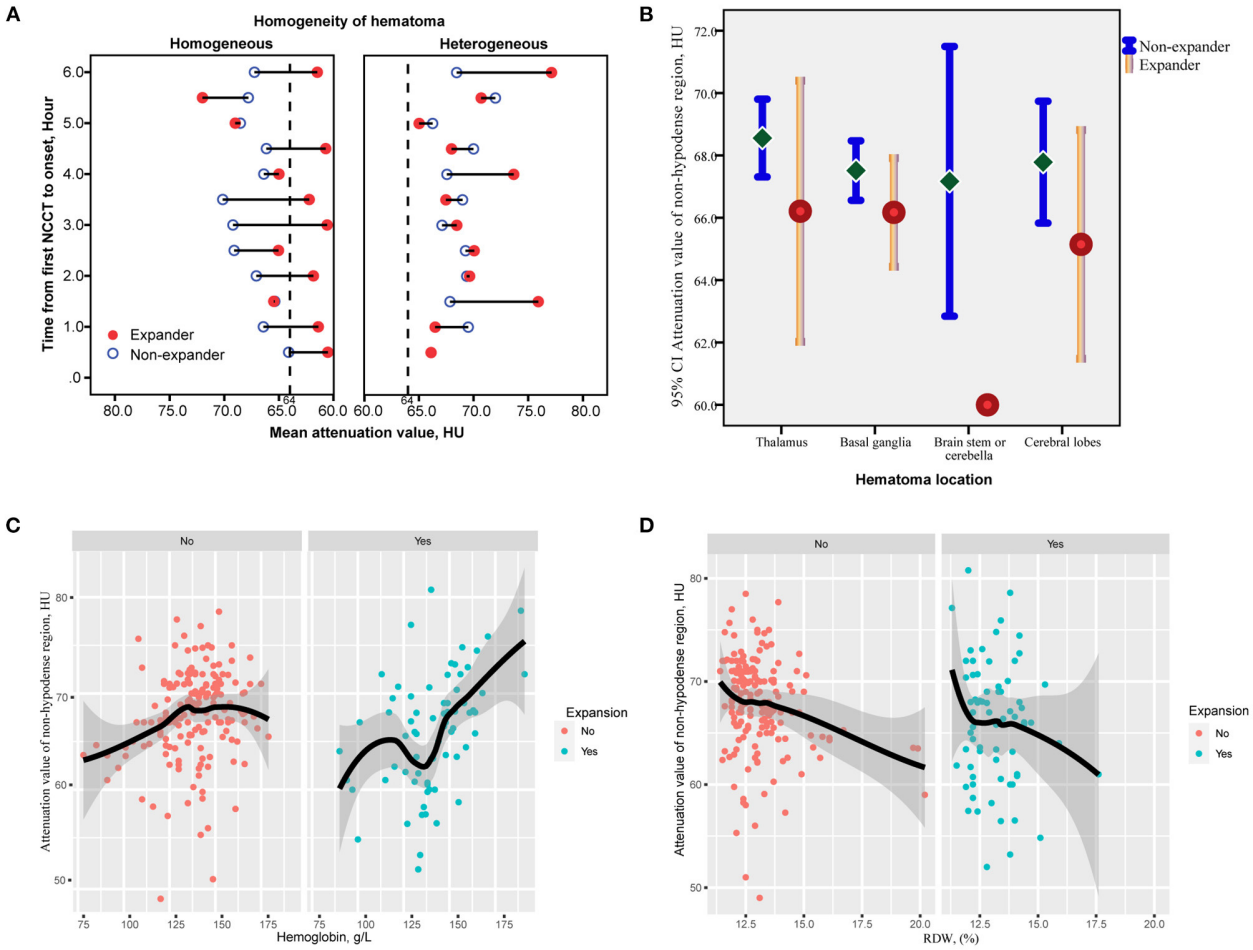
Correlation of the attenuation value of non-hypodense region and time from the first NCCT to onset between hematomas with or without hypodensities. **(A)** The attenuation value of the non-hypodense region of most expanded hematomas remains lower irrespective of time prolonging, whereas no trend exists between the time and non-hypodense region attenuation value of heterogeneous hematomas. **(B)** The mean value and 95% *CI*s of the attenuation value of the non-hypodense region at different cerebral locations. The attenuation value of the non-hypodense region of the cerebellar and brainstem hematomas is the lowest and differs most significantly between expanders and non-expanders. **(C,D)** Loess analysis of attenuation value and hemoglobin and RDW between expanders and non-expanders. The attenuation value increases with a higher hemoglobin level, but with a lower RDW. HU, Hounsfield units; RDW, red blood cell distribution width.

The correlation matrix heatmap for continuous variables are shown in Supplementary Figure 2. In homogeneous hematomas, the attenuation value within the core region was relatively low in those with HE compared with those without HE, and without a significant increasing trend over time (Figure 4A). In heterogeneous density hematomas, there was no significant correlation between the attenuation value within the non-hypodense region of the hematoma and the time from the first NCCT scan to onset, irrespective of the occurrence of HE (Figure 4A). NCCT attenuation value was generally lower within the non-hypodense region of expanded hematomas, and this difference was more pronounced in the brainstem or cerebellar locations (Figure 4B). A positive correlation existed between attenuation value within the non-hypodense region and hemoglobin level (Figure 4C), and a negative correlation existed referring to the red blood cell distribution width, regardless of whether the hematoma was expanded (Figure 4D).

### Derivation of the Critical Attenuation Value of Hematoma to Predict HE

**Figure 5:**
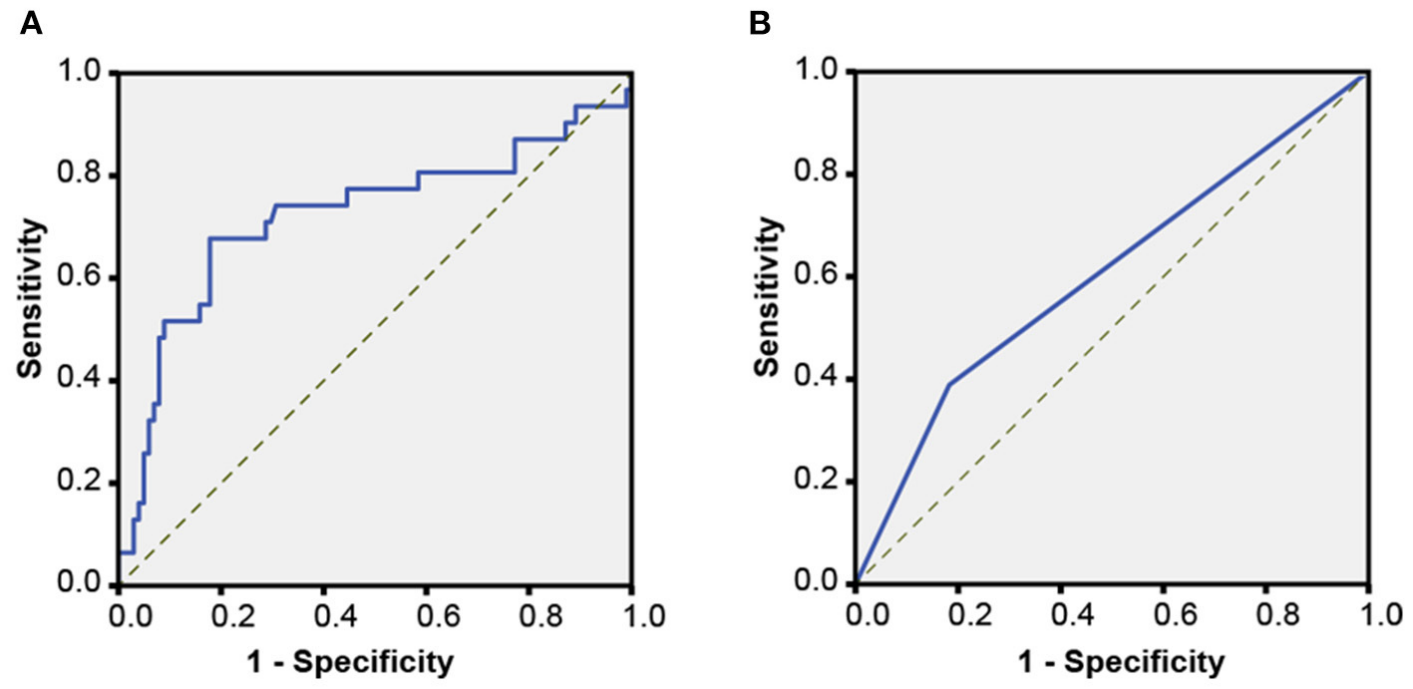
Receiver operating characteristic (ROC) curves. **(A)** An ROC analysis for determining the critical attenuation value of the non-hypodense region of hematoma in the derivation cohort. **(B)** An ROC analysis of the attenuation value <64 HU for predicting HE in the validation cohort.

An ROC curve analysis showed that the cutoff of attenuation value within non-hypodense region for predicting HE was <64 HU, and its sensitivity, specificity, PLR, NLR, PPV, NPV, and AUC were 67.74% (95% *CI*, 48.6–83.3%), 82.18% (95% *CI*, 73. 3–89.1%), 3.8 (95% *CI*, 2.3–6.2), 0.39 (95% *CI*, 0.2–0.7), 53.8% (95% *CI*, 41.8–65.4%), 89.2% (95% *CI*, 83.2–93.3%), and 0.722 (95% *CI*, 0.600–0.844, *p* < 0.001), indicating its suitability for predicting HE (Figure 5A). A total of 40 hematomas had an attenuation value of <64 HU within the non-hypodense region and there were 31 heterogeneous hematomas. Among them, 8 of 9 hematomas with regular morphology and homogeneous density and suffered HE, and had a density value of <64 HU (*p* < 0.001). Seven of the 11 patients with irregular morphology, homogeneous density, and dilatation had an attenuation value < 64 HU (*p* = 0.028). Among the heterogeneous hematomas, 6 of the 9 hematomas with a non-hypodense region attenuation value of <64 HU and only 5 of the 22 hematomas with a non-hypodense region attenuation value of >64 HU that eventually expanded (*p* = 0.02).

### Validation of the Association Between Attenuation Value < 64 HU and HE

There was good inter-rater (rater 1, κ = 0.967; rater 2, κ = 0.956) and intra-rater (κ = 0.945, rater 1 vs. rater 2) agreement for the measurement of attenuation value <64 HU. A univariate logistic analysis showed that attenuation value within non-hypodense region <64 HU was associated with HE, both unadjusted and after adjusting for other factors (Table 2). An ROC analysis showed that the sensitivity, specificity, PLR, NLR, PPV, NPV, and AUC of the attenuation value <64 HU for HE prediction were 36.11% (95% *CI*, 25.1–48.3%), 81.71% (95% *CI*, 75.2–87.1%), 1. 97 (95% *CI*, 1.3–3.1), 0.78 (95% *CI*, 0.6–0.9), 44.8% (95% *CI*, 34.4–55.8%), 75.7% (95% *CI*, 72.0–78.9%), and 0.589 (95% *CI*, 0.526–0.652, *p* = 0.005) (Figure 5B). There were 89 heterogeneous hematomas and there were 58 hematomas that had attenuation value < 64 HU within the non-hypodense region. Of the 14 hematomas with regular morphology, homogenous density and subsequent expansion, 9 had attenuation value <64 HU (*p* < 0.001). Out of 22 patients with irregular morphology, homogeneous density and who underwent expansion, 11 had attenuation value < 64 HU (*p* = 0.041). Among heterogeneous hematomas, expansion occurred in the 6 of 12 hematomas with attenuation value <64 HU in the non-hypodense region and in 30 of 77 hematomas with attenuation value >64 HU in the non-hypodense region of the hematoma (*p* = 0.683).

**Table 2:** Variables with statistical significance for predicting HE analyzed by univariate logistic regression.

**Variables**	**Crude**			**Model 1**			**Model 2**			**Model 3**		
	**OR**	**95% CI**	***P***	**OR**	**95% CI**	***P***	**OR**	**95% CI**	***P***	**OR**	**95% CI**	***P***
Sex, male	2.056	1.103–3.834	0.023	2.033	1.028–4.022	0.041	2.235	0.948–5.272	0.066	3.140	1.129–8.734	0.028
Time from first NCCT scan to onset, hour	0.764	0.632–0.925	0.006	0.751	0.618–0.914	0.004	0.673	0.532–0.850	0.001	0.611	0.462–0.809	0.001
Baseline GCS score	1.562	1.100–2.219	0.013	1.644	1.140–2.373	0.008	1.470	0.966–2.236	0.072	1.297	0.762–2.208	0.338
Baseline hematoma volume, ml	1.023	1.010–1.037	0.001	1.024	1.011–1.038	<0.001	1.022	1.006–1.038	0.008	1.014	0.993–1.037	0.200
Blend sign	2.980	1.559–5.695	0.001	2.860	1.462–5.595	0.002	3.916	1.793–8.552	0.001	3.471	1.326–9.038	0.011
Irregular sign	3.223	1.825–5.691	<0.001	3.578	1.957–6.540	<0.001	3.554	1.739–7.263	0.001	2.911	1.021–8.304	0.046
Island sign	1.727	1.118–2.668	0.014	1.948	1.219–3.115	0.005	2.227	1.303–3.808	0.003	2.079	0.954–4.527	0.065
Attenuation value of non-hypodense region, HU	0.935	0.886–0.986	0.014	0.926	0.876–0.979	0.007	0.912	0.854–0.974	0.006	0.845	0.770–0.928	<0.001
Attenuation value of non-hypodense region <64 HU	2.140	1.111–4.122	0.023	3.072	1.577–5.986	0.001	4.337	1.885–9.978	0.001	10.252	3.306–31.788	<0.001

*Variables that were statistically significant in the univariate analysis were adjusted again to observe whether there was a significant change of OR for the coexistence of other factors*.

*Model 1: Adjusted for age, sex, co-existing diseases (hypertension, diabetes, current smoker, current alcohol drinker, and stroke history), and antiplatelets or anticoagulants therapy*.

*Model 2: Adjusted for Model 1, physical examination results (systolic pressure, diastolic pressure, and baseline GCS score) and laboratory findings (RBC count, hemoglobin, hematocrit, MCV, MCH, MCHC, RDW, platelet count, platelet distribution width, prothrombin time, activated partial thromboplastin time, international normalized ratio, total cholesterol, and serum glucose)*.

*Model 3: Adjusted for Model 2, time from the first NCCT scan to onset, and hematoma features on NCCT scan (location, baseline hematoma volume, hematoma width/length ratio on axial section, midline shift distance > 0.5 cm, subarachnoid hemorrhage, intraventricular hemorrhage, swirl sign, black hole sign, blend sign, irregular sign, satellite sign, and island sign)*.

*HE, hematoma expansion; OR, odds ratio; CI, Confidence interval; NCCT, non-contrast CT; GCS, Glasgow coma scale; HU, Hounsfield units; RBC, red blood count; MCV, mean corpuscular volume; MCH, mean corpuscular hemoglobin; MCHC, mean corpuscular hemoglobin concentration; RDW, RBC distribution width*.

**Figure 6:**
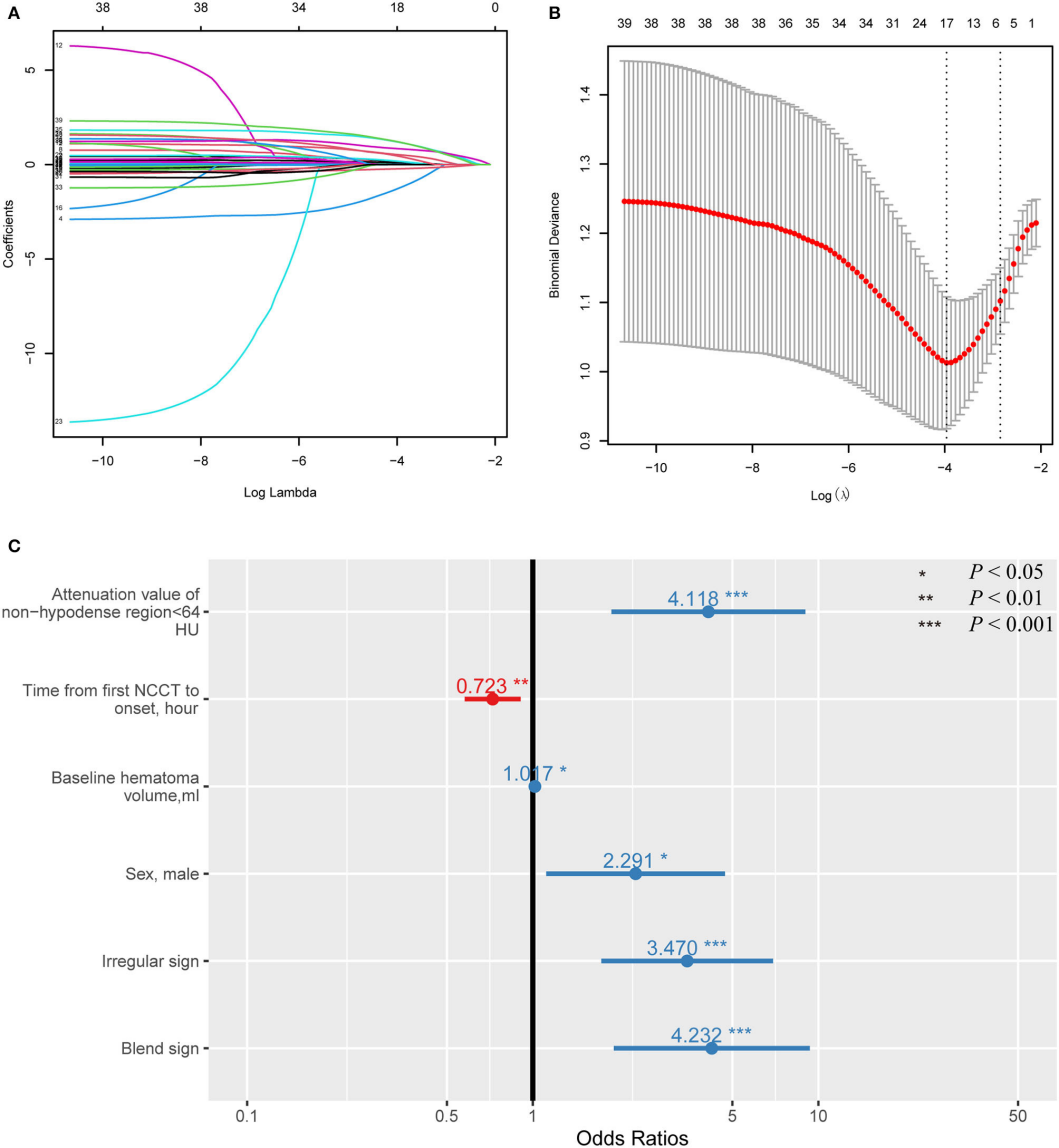
Forest plot showing the results of multivariate analysis. The potential predictors included in the multivariate analysis were first screened by the least absolute shrinkage and selection operator (LASSO) regression. **(A)** LASSO coefficient curves for 39 variables, such as age, sex, time of onset, radiological characteristics of the hematoma, medical history, physical examination, and laboratory parameters. **(B)** Dotted vertical lines were drawn at the optimal values by using the minimum criteria and the one-standard error of the minimum criteria (the 1-SE criteria). Six variables with non-zero coefficients (attenuation value of non-hypodense region <64 HU, time from the first NCCT scan to onset, baseline hematoma volume, sex, irregular sign, blend sign) were identified. **(C)** Forest plot of all six variables identified by LASSO regression that have *p* < 0.05.

A LASSO analysis was applied to screen for predictors without the collinearity of HE (Figures 6A,B). The multivariate model showed that an attenuation value <64 HU remained an independent predictor [odds ratio (*OR*), 4.118; 95% *CI*, 1.897–9.129, *p* < 0.001] after adjusting for male sex, time from the first NCCT scan to onset, baseline hematoma volume, blend sign, and irregular sign (Figure 6C, Supplementary Table 1).

**Figure 7:**
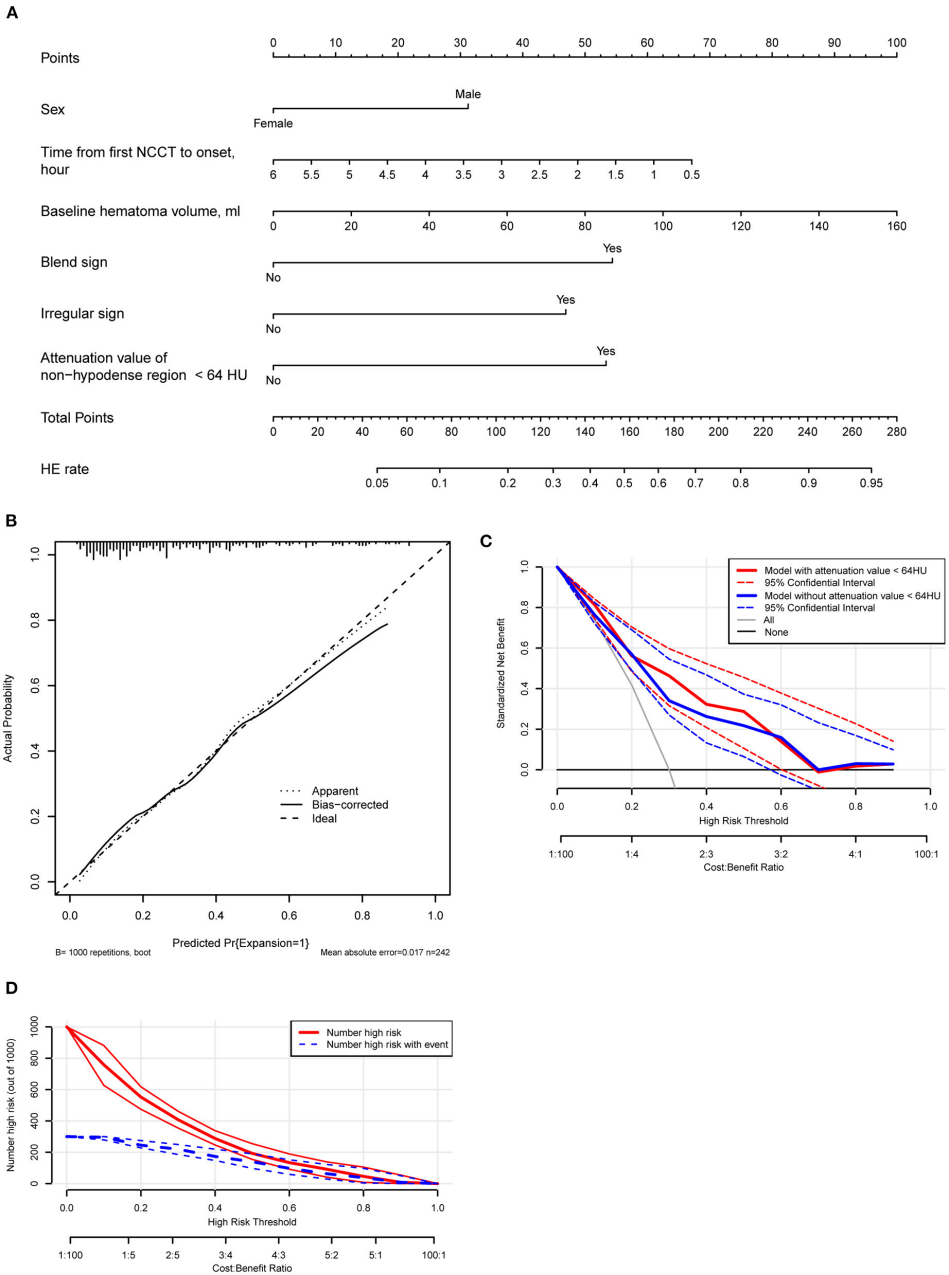
**(A)** A nomogram is derived from the multivariate analysis. **(B)** Calibration plot of the nomogram model. **(C)** A decision curve analysis of the model with or without incorporation of the attenuation value of the non-hypodense region of hematoma. The model with six variables has a higher net benefit when the expansion risk threshold varies between 0.3 and 0.6. **(D)** Clinical impact curve analysis. The actual events and the predicted events are close while the risk of threshold exceeds about 0.5.

Based on the multivariate model, the nomogram was constructed (Figure 7A) and its discriminating and calibrating ability was favorable with a C-index/AUC of 0.806 (Supplementary Figure 3) and good calibration (Figure 7B). In addition, the model showed good discrimination ability in derivation cohort and combined cohorts with the C-indexes of 0.883 and 0823. A decision curve analysis showed that an attenuation value < 64 HU significantly improved the predicted net benefit when the probability of HE varied in the range of ~0.3–0.6 (Figure 7C). The clinical impact curve showed that the predicted and actual number of HE was close when the threshold risk of HE exceeded ~0.5 (Figure 7D).

## Discussion

The attenuation value of intracerebral hematoma on the NCCT is one of the indicators that have received much attention in recent years for it can be used to predict HE which seriously affects the prognosis of a patient (14–16). Studies had shown that the difference of attenuation value between high-density and low-density areas that had a clear margin within hematoma > 18 HU (7, 17–19), and the minimal attenuation value of the hematoma ≤ 31 HU were independent risk factors for predicting HE (20). Here, according to our study, the attenuation value within the non-hypodense region of the hematoma is also an independent predictor associated with HE, and an attenuation value <64 HU is a potential cutoff.

There is no doubt that the hemostatic status of the bleeding site of the ruptured vessel is the fundamental cause of whether the hematoma will enlarge. In the early stages of intracerebral hemorrhage, the density of fresh hematoma is ~+30 to 45 HU (21). Then, it gradually increases during the initial ~48 h and then decreases again after reaching the peak value (5, 22, 23). The increased attenuation value of the hematoma is mainly due to the formation of a meshwork of fibrin fibers, globin molecules, and early clot contraction events after bleeding (5, 24), therefore hypodense foci within the hematoma may be the result of repeated bleeding from the primary bleeding site or poor clot contraction and hence the evidence of the potential HE (7, 9, 17). However, the exact site of hemorrhage, whether it is a primary hemorrhagic vessel (25), or a secondary hemorrhagic vessel (26), is not always within the hypodense foci, but may also be located within the non-hypodense region and appear as a spot sign on contrast CT (6, 27). Therefore, the role played by the attenuation value within the non-hypodense region in determining the hemostatic status of the bleeding site is not negligible.

The attenuation value of the hyperdense area of hematoma has been taken into count sparsely in assessing the probability of HE. In the acquirement of the mean attenuation value of hematoma by Jeong et al., both the hypodense and non-hypodense regions of hematoma were measured in a *post-hoc* analysis, though the mean density of the hematoma with and without hypodense foci was not statistically significant, the mean density of the dilated hematoma was significantly lower (5). Nevertheless, their study failed to account for the proportion of high-density and low-density regions in each hematoma, so the unique role of the non-hypodense regions in hematoma expansion cannot be accurately determined. In contrast, our study confirmed that the non-hypodense regions of the expanded hematoma do possess a lower attenuation value. To the best of our knowledge, there is no clearly reported indicator regarding NCCT hematoma density that can be used as a predictor of secondary expansion of homogeneous hematomas. Since the existing hypodensity sign, swirl sign, black hole sign, and blend sign are based on hypodense regions to determine the risk of HE, the attenuation value within the non-hypodense region of the hematoma provides an option to determine whether a homogeneous hematoma is at high risk. In addition, heterogeneous hematomas with this feature may have a greater likelihood of expansion. This feature does not intersect with the hematoma morphology and low-density markers that predict HE, and quantifies the density values of the high-density areas independently of the low-density areas within the hematoma, avoiding the influence of the proportion of low-density areas in the calculation of mean density value and thus having independent diagnostic value.

The multifactor model incorporated predictors involving the attenuation value level within the non-hypodense region that was determined by LASSO regression and was presented in the form of a nomogram, allowing for a clearer understanding of the role played by each predictor. The nomogram is simple and feasible, applicable to individual patients and practitioners in daily clinical practice, and the data are easily accessible (28, 29). The discriminative power of our model is good compared with previous reports (29, 30), with a cutoff nomogram score of about 126 and a corresponding prevalence of about 0.3 of expansion, the patient has a high probability of HE. A decision curve analysis can be used to visually and graphically evaluate the ability of each component to improve the model and has been highly recommended in recent years (31, 32). Our model shows that the net benefit of treatment is higher when the risk of hematoma varies between 0.3 and 0.6, thus taking into account the magnitude of the attenuation value within the non-hypodense region may be more helpful (Net Reclassification Improvement = 0.4336, *p* = 0.001; Integrated Discrimination Improvement = 0.0483, *p* = 0.002) in making the right clinical decisions when the risk of HE determined by assessment methods is within this range. Besides, the prediction will be more accurate if the threshold of HE is >0.5 according to the clinical impact curve.

The study has some limitations. First, the relatively small number of subjects may have produced some selective bias. In addition, the attenuation of the non-hypodense region was not suitable for predicting intracerebral hematomas with volume <1 ml, which means that most hematomas located in the midbrain, pons, and medulla may not be applicable. The study also excluded multiple intracerebral hemorrhages, although some of these hematomas may have the same etiology, the non-hypodense region attenuation value, and associated model may not be suitable for these hematomas. Again, although the model performed well in our dataset, a follow-up replication study is necessary to validate it as the new metric that has not been studied in other literature. Finally, the optimal cutoff of the new predictor may need to be modified to reach higher accuracy.

## Conclusions

In conclusion, our study explored and validated the attenuation value of the non-hypodense region of hematoma that was independently associated with early HE in patients with spontaneous cerebral hemorrhage. The critical attenuation value < 64 HU was shown to be an appropriate indicator of possible subsequent HE and was able to significantly improve the predictive power of the multifactor model.

## Data Availability Statement

The raw data supporting the conclusions of this article will be made available on request to the corresponding author, without undue reservation.

## Ethics Statement

The studies involving human participants were reviewed and approved by Ethics Committee of Tongji Hospital, Huazhong University of Science and Technology. Written informed consent for participation was not required for this study in accordance with the national legislation and the institutional requirements.

## Author Contributions

YC contributed to the study conception and design. Material preparation, data collection, and analysis were performed by YC, DC, X-LM, and Z-QG. The first draft of the manuscript was written by YC and reviewed by Y-BO, YH, XC, and JC. All authors commented on previous versions of the manuscript. All authors read and approved the final manuscript.

## Conflict of Interest

The authors declare that the research was conducted in the absence of any commercial or financial relationships that could be construed as a potential conflict of interest.

## Publisher's Note

All claims expressed in this article are solely those of the authors and do not necessarily represent those of their affiliated organizations, or those of the publisher, the editors and the reviewers. Any product that may be evaluated in this article, or claim that may be made by its manufacturer, is not guaranteed or endorsed by the publisher.

## Supplementary Material

Supplementary Figure 1 Flowchart for the patient selection of two patient cohorts.

Supplementary Figure 2 Correlation heatmap of the potential predictors (continuous variables) of hematoma expansion (HE). The hemoglobin, hematocrit, and MCHC are the positively related factor of HU, whereas the RDW is the negatively related factor that with statistical significance. SBP, systolic pressure; DBP, diastolic pressure; RBC, red blood cell; Hb, hemoglobin; HCT, hematocrit; MCV, mean corpuscular volume; MCH, mean corpuscular hemoglobin; MCHC, mean corpuscular hemoglobin concentration; RDW, RBC distribution width; PLT, platelet; PDW, platelet distribution width; PT, prothrombin time; APTT, activated partial thromboplastin time; INR, international normalized ratio; TC, total cholesterol; SG, serum glucose; BV, baseline hematoma volume; HU, Hounsfield units.

Supplementary Figure 3 The receiver operating characteristic (ROC) analysis of the multivariate model. The C-index/area under the curve (AUC) is 0.806. The corresponding nomogram score for cutoff is 126 (asterisk).

Supplementary Table 1 Multivariate analysis of the predictors of HE identified by LASSO regression.

## Abbreviations

NCCT: non-contrast computed tomography
HE: hematoma expansion
ROC: receiver operating characteristic
LASSO: least absolute shrinkage and selection operator
HU: Hounsfield units
PPV: positive predictive value
NPV: negative predictive value
PLR: positive likelihood ratio
NLR: negative likelihood ratio
AUC: area under the curve
IQR: interquartile range.
