# Supplementary figures and images for: The Attenuation Value Within the Non-hypodense Region on Non-contrast Computed Tomography of Spontaneous Cerebral Hemorrhage: A Long-Neglected Predictor of Hematoma Expansion
Source: Front Neurol. 2022 Apr 8;13:785670. doi: 10.3389/fneur.2022.785670 (PMC9024072; doi:10.3389/fneur.2022.785670)

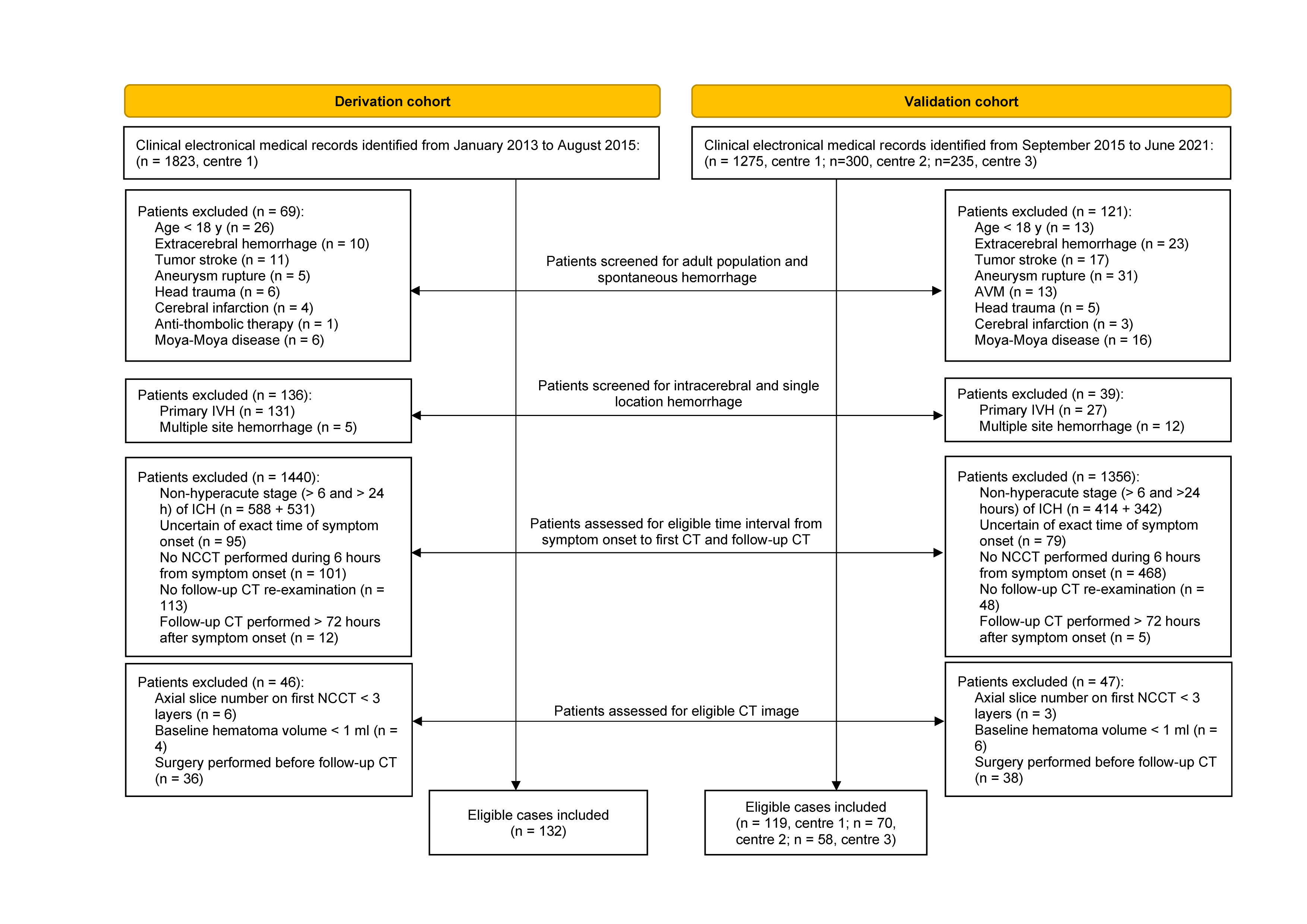

Supplement: Supplementary Figure 1 — Flowchart for the patient selection of two patient cohorts. [file Image_1.TIF]

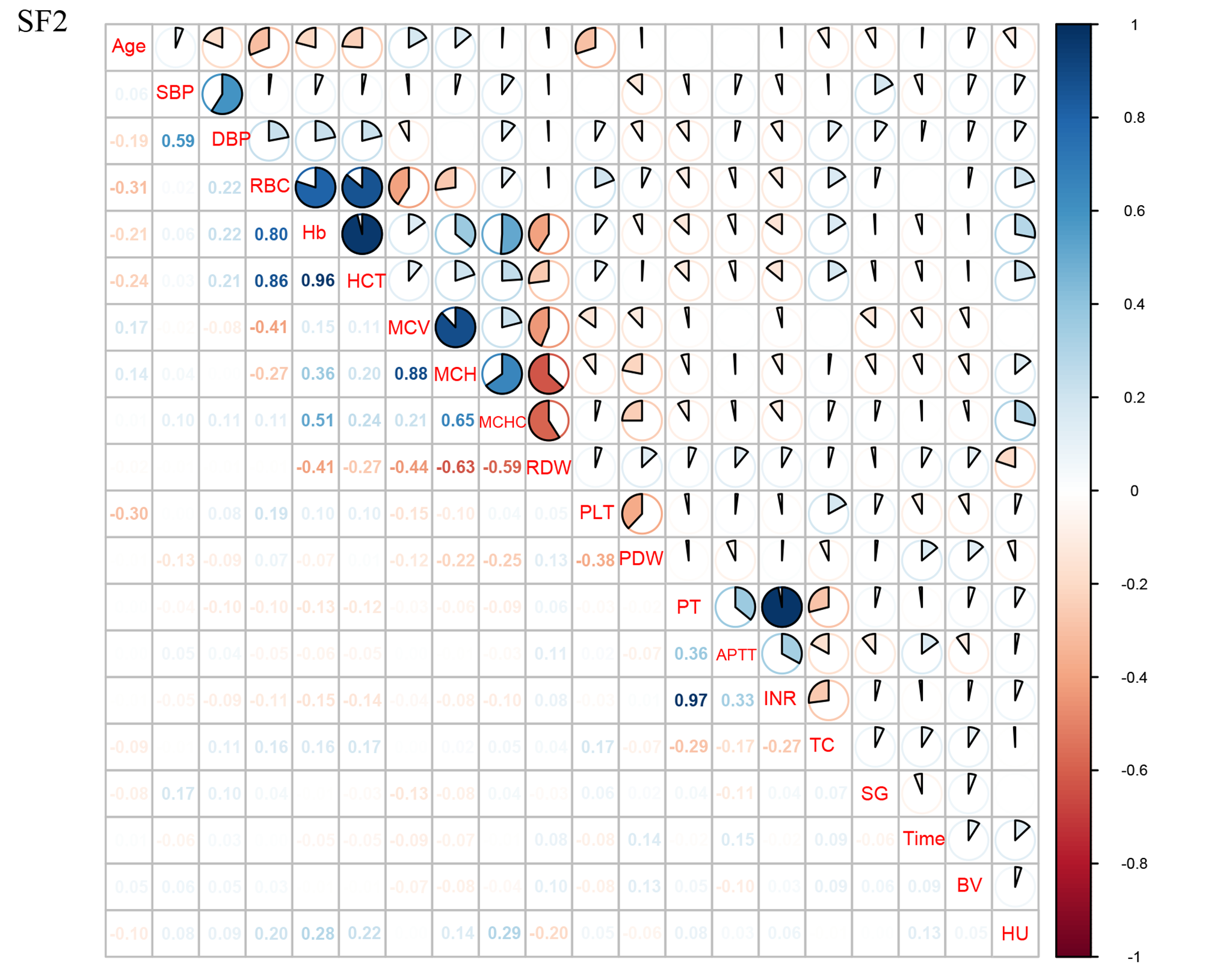

Supplement: Supplementary Figure 2 — Correlation heatmap of the potential predictors (continuous variables) of hematoma expansion (HE). The hemoglobin, hematocrit, and MCHC are the positively related factor of HU, whereas the RDW is the negatively related factor that with statistical significance. SBP, systolic pressure; DBP, diastolic pressure; RBC, red blood cell; Hb, hemoglobin; HCT, hematocrit; MCV, mean corpuscular volume; MCH, mean corpuscular hemoglobin; MCHC, mean corpuscular hemoglobin concentration; RDW, RBC distribution width; PLT, platelet; PDW, platelet distribution width; PT, prothrombin time; APTT, activated partial thromboplastin time; INR, international normalized ratio; TC, total cholesterol; SG, serum glucose; BV, baseline hematoma volume; HU, Hounsfield units. [file Image_2.TIF]

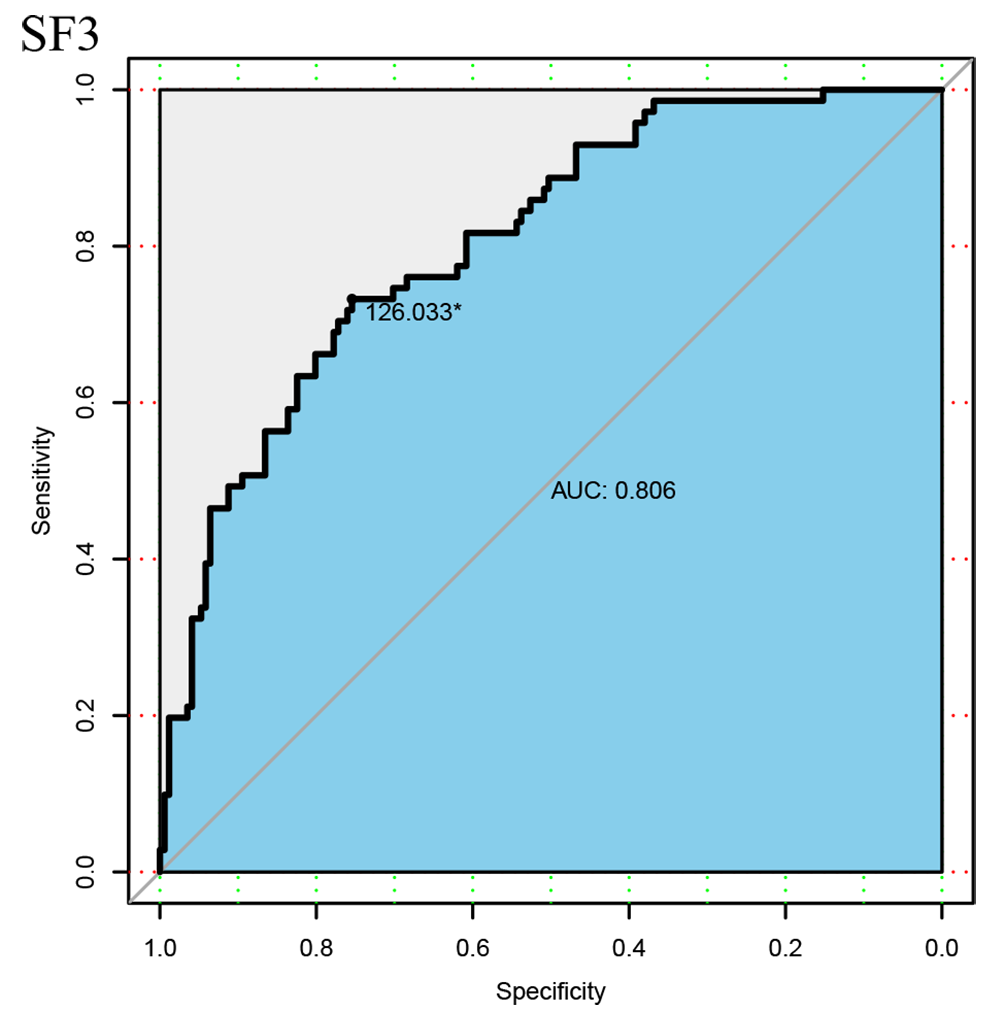

Supplement: Supplementary Figure 3 — The receiver operating characteristic (ROC) analysis of the multivariate model. The C-index/area under the curve (AUC) is 0.806. The corresponding nomogram score for cutoff is 126 (asterisk). [file Image_3.TIF]
