# Supplementary material for: The Attenuation Value Within the Non-hypodense Region on Non-contrast Computed Tomography of Spontaneous Cerebral Hemorrhage: A Long-Neglected Predictor of Hematoma Expansion
Source: Front Neurol. 2022 Apr 8;13:785670. doi: 10.3389/fneur.2022.785670 (PMC9024072; doi:10.3389/fneur.2022.785670)
Supplement: Supplementary Table 1 — Multivariate analysis of the predictors of HE identified by LASSO regression. [file Table_1.docx]

Supplementary Table 1. Multivariate analysis of the predictors of HE identified by LASSO regression.

| **Variables** | **OR** | **95% CI** | **p** |
| --- | --- | --- | --- |
| **Attenuation value of non-hypodense region < 64 HU** | 4.118 | 1.897-9.129 | <0.001 |
| **Time from first NCCT scan to onset, hour** | 0.723 | 0.573-0.900 | 0.005 |
| **Baseline hematoma volume, ml** | 1.017 | 1.001-1.033 | 0.037 |
| **Sex, male** | 2.291 | 1.135-4.828 | 0.024 |
| **Irregular sign** | 3.470 | 1.756-7.046 | <0.001 |
| **Blend sign** | 4.231 | 1.933-9.444 | <0.001 |

The C-index of the model is 0.806.

Abbreviations: HE, hematoma expansion; LASSO, least absolute shrinkage and selection operator; OR, odds ratio; CI, confidential interval; HU, Hounsfield units, NCCT, non-contrast CT.
